# Development and psychometric validation of a tool to assess healthcare providers' knowledge, attitudes, and practices in pediatric injury rehabilitation in Northern Tanzania

**DOI:** 10.3389/fresc.2026.1960075

**Published:** 2026-10-26

**Authors:** Baraka Moshi, Blandina T. Mmbaga, Elizabeth M. Keating, Catherine A. Staton, Haleluya Moshi, Michael J. Mahande, Joao Ricardo Nickenig Vissoci

**Affiliations:** 1 Kilimanjaro Clinical Research Institute, Moshi, Tanzania; 2 Department of Epidemiology & Biostatistics, School of Public Health, KCMC University, Moshi, Tanzania; 3 Global Emergency Medicine Innovation and Implementation (GEMINI) Research Centre, Duke Global Health Institute, Duke University, Durham, NC, United States; 4 Duke Global Health Institute, Duke University, Durham, NC, United States; 5 Department of Pediatric and Child Health, School of Medicine, KCMC University, Moshi, Tanzania; 6 Department of Pediatrics, Division of Pediatric Emergency Medicine, University of Utah, Salt Lake City, UT, United States; 7 Department of Emergency Medicine, Duke University Medical Center, Durham, NC, United States; 8 Kilimanjaro Christian Medical Centre, Moshi, Tanzania; 9 Department of Physiotherapy, School of Rehabilitation Medicine, KCMC University, Moshi, Tanzania

**Keywords:** health workforce, knowledge, attitudes and practices, pediatric injury rehabilitation, psychometrics, Tanzania, tool validation

## Abstract

**Background:**

Rehabilitation is essential for improving outcomes in pediatric injury care, yet its effectiveness depends on healthcare providers’ knowledge, attitudes, and practices (KAP). However, no standardized tool exists to assess these factors in pediatric injury rehabilitation. This study aimed to develop and validate a tool assessing healthcare providers’ KAP in pediatric injury rehabilitation in Northern Tanzania.

**Methods:**

A methodological study with cross-sectional validation was conducted among multidisciplinary healthcare providers involved in pediatric injury rehabilitation. The KAP tool was initially developed through a literature review and methodological guidance, followed by formal content evaluation by five experts and pilot testing with 10 healthcare providers. Participant characteristics and item responses were summarized using descriptive statistics. Exploratory Factor Analysis (EFA) was conducted using a sample of 102 healthcare providers, followed by Confirmatory Factor Analysis (CFA) in a separate sample of 210 healthcare providers. Content validity, construct validity, and internal consistency were assessed using the Content Validity Index, factor analysis, and reliability coefficients, respectively.

**Results:**

The final tool included 42 items across knowledge (23), attitude (9), and practice (10) domains, reduced from an initial 72 items, content validity was strong (CVI = 0.88). EFA supported a three-factor structure for the knowledge domain and single-factor structures for the attitude and practice domains. CFA further supported the proposed structure, demonstrating acceptable model fit across all domains. Internal consistency ranged from acceptable to good, with Cronbach's alpha coefficients ranging from 0.78 to 0.88 and McDonald's omega ranging from 0.79 to 0.89.

**Conclusions:**

The validated KAP tool demonstrates acceptable psychometric properties and can be used to assess healthcare providers’ competencies in pediatric injury rehabilitation. It provides a standardized instrument for research and program evaluation in similar resource-limited settings.

## Introduction

Pediatric injuries are a major cause of morbidity and mortality worldwide, with a disproportionately high burden in Low and middle income countries (LMIC's) (1). In Tanzania, a study across 13 health facilities found that nearly one-quarter (23.5%) of trauma patients were children, with falls (36.5%) and road traffic crashes (19.2%) being the most common causes (2). Rehabilitation services play a critical role in restoring function, improving quality of life, and facilitating social reintegration for children with injuries (3). These services, provided by healthcare professionals, help children recover from injury, regain mobility, perform daily activities and participate in their communities.

Despite its importance, access to pediatric rehabilitation in Northern Tanzania remains limited. A systematic review in the Kilimanjaro region identified eleven organizations providing rehabilitation services, though only a few offered comprehensive care and financial accessibility was limited. While a range of services exists, there remains a significant gap in connecting patients to long-term rehabilitation, reflecting broader challenges in low- and middle-income settings where many individuals in need do not receive adequate care (4). In addition to structural barriers, the quality of rehabilitation services is influenced by healthcare providers' knowledge, attitudes, and practices (KAP). Effective rehabilitation depends on providers who are adequately trained, motivated, and able to deliver consistent, evidence-based care (5). However, in resource limited settings like Tanzania, these aspects are often poorly understood and inadequately assessed (6).

Numerous KAP instruments have been developed across healthcare domains, including chronic disease management (7), snakebite and emergency care preparedness in low-resource settings (8) and nursing care practice (9). These tools often demonstrate strong psychometric properties and have informed training and policy. However, they are typically condition-specific, developed in high-income settings, or focused on single professional groups. None address multidisciplinary providers involved in pediatric injury rehabilitation in low-resource settings.

KAP surveys are commonly used in healthcare research to assess healthcare providers' knowledge, attitudes, and practices regarding specific domains (10). They are quite familiar to implement, adaptable to a wide range of topics and populations, and when well-designed can provide valuable baseline or monitoring data to inform training programs, policy development, and service delivery planning. The cost of conducting such surveys is generally modest compared with larger-scale interventions (11). During the COVID-19 pandemic, KAP surveys highlighted value in shaping rapid health system responses to emerging challenges (12). KAP surveys assist in identifying knowledge gaps, misconceptions, and suboptimal practices that may impact the effectiveness of rehabilitation interventions. Furthermore, KAP surveys can be tailored to address specific topics or populations, such as healthcare providers' practices in rehabilitation to pediatric injuries, thus offering a practical and adaptable tool for improving healthcare service quality and guiding future training or policy adjustments.

Despite their utility, no standardized and psychometrically validated tool exists to assess healthcare providers' KAP in pediatric injury rehabilitation in Tanzania or similar low- and middle-income settings. This limits the ability to identify training needs, evaluate service readiness, and guide evidence-informed improvements. Therefore, this study aimed to develop and evaluate the psychometric properties of a KAP survey assessing healthcare providers involved in the rehabilitation of children following injury at a zonal referral hospital in Northern Tanzania. This tool is intended to support service evaluation, professional training, and research in pediatric rehabilitation in resource-limited settings.

## Methods

### Study design

This was a methodological study with cross-sectional field validation conducted in four phases: (1) item generation, (2) content validation, (3) pilot testing, and (4) field testing for psychometric evaluation.

### Participant recruitment and data collection period

Participant recruitment and data collection for the KAP tool validation study were conducted between 15/01/2025 and 15/12/2025.

### Study setting

KCMC is a zonal referral hospital in north-eastern Tanzania, serving an estimated catchment population of 15 million people from both urban and rural areas. The hospital provides rehabilitation services relevant to this study through its physiotherapy, occupational therapy, speech and language therapy, optometry, and prosthetics/orthotics units, offering both inpatient and outpatient care. The Emergency Medical Department (EMD) sees approximately 1,400–1,700 pediatric injury cases annually (13), providing an appropriate setting for studying pediatric rehabilitation needs.

### Study population

The study included three groups of participants at different stages of instrument development and validation. First, five experts were purposively selected to evaluate the content of the initially developed questionnaire. The questionnaire was initially developed by the Corresponding Author based on a review of the relevant literature and methodological guidance, with ongoing input and guidance from his three PhD supervisors and four mentors involved in the research. The expert panel comprised a methodologist with expertise in scale development and psychometrics, a pediatrician with expertise in pediatric injury care and clinical management, a physiotherapist with expertise in pediatric physical rehabilitation, an occupational therapist with expertise in functional rehabilitation, and a prosthetist/orthotist with expertise in pediatric prosthetic and orthotic rehabilitation. The experts reviewed the questionnaire for the relevance, clarity, comprehensiveness, and appropriateness of its items. They were selected based on their relevant expertise and experience to provide complementary methodological, pediatric clinical, and rehabilitation perspectives, rather than to represent all rehabilitation professions. Although the panel included professionals from key areas relevant to the instrument, some disciplines, including speech and language therapy and psychology, were not represented. This was partly due to the limited availability of these professionals in the study setting, where only one speech and language therapist and one psychologist were available. Their absence from the expert panel is acknowledged as a potential limitation of the content-validation process. Second, 10 healthcare providers participated in pilot testing. These included two physiotherapists, one occupational therapist, one prosthetist, one orthotist, one optometrist, one orthopaedic surgeon, one nurse, one emergency physician, and one pediatrician. The pilot participants were selected from healthcare providers involved in the care and rehabilitation of children following injury. Their role was to assess the clarity, comprehensibility, relevance, and applicability of the questionnaire items and to identify items requiring clarification before field testing.

Third, 102 healthcare providers participated in the initial field-testing phase, and their data were used for Exploratory factor analysis (EFA). A separate sample of 210 healthcare providers subsequently participated in the Confirmatory factor analysis (CFA) phase. All participants were involved in the rehabilitation management of children following injury. The professional composition of the 102 and 210 participants is presented in Tables 2a,b, respectively.

Overall, the intended target population of the instrument was healthcare providers involved in the rehabilitation of children following injury. The instrument was designed to assess core knowledge, attitudes, and practices relevant to pediatric injury rehabilitation rather than profession-specific competencies of any single rehabilitation discipline.

### Sample size and sampling technique

Sampling was conducted in three phases. In the first phase, an expert panel comprising five individuals was purposively selected to provide formal content evaluation of the initially developed questionnaire. This approach is consistent with established methodological guidance recommending the use of a small, purposively selected group of knowledgeable experts for in-depth evaluation of instrument content at the item level (14). This composition allowed for comprehensive appraisal of the questionnaire across clinical, rehabilitative, and methodological dimensions. The use of a small, purposefully selected expert panel is consistent with established methodological guidance for content and face validation of KAP questionnaires, where expert evaluation is typically undertaken by a limited number of knowledgeable reviewers to ensure in-depth item-level assessment of relevance, clarity, and appropriateness (15–17). Based on the experts' evaluation, the questionnaire was revised and refined to address issues related to the relevance, clarity, comprehensiveness, and appropriateness of the items. In the second phase, 10 healthcare providers involved in pediatric injury care and rehabilitation were purposively selected to pilot the revised questionnaire. The pilot study assessed the clarity, comprehensibility, relevance, and applicability of the questionnaire from the perspective of the intended users and helped identify any remaining issues requiring clarification before field testing. In the third phase, construct validity was assessed in two stages. EFA was first conducted using data from 102 healthcare providers, followed by CFA using data from a separate sample of 210 healthcare providers recruited for field validation. Although psychometric literature often suggests larger samples for factor analysis, sample size adequacy is not determined by a single fixed number and depends on several factors, including model complexity, number of items, factor loadings, and overall data quality (18). In addition, methodological studies have shown that commonly used “rules of thumb” for sample size in factor analysis are not strongly supported by empirical evidence and should not be applied rigidly (19). In this study, the sample size was also guided by feasibility and the available number of eligible healthcare providers in the study setting. Similar studies on instrument development have used small sample sizes compare to current study; for example, research conducted in Pakistan validating a Chronic Kidney Disease Knowledge, Attitude, and Practice tool included 100 practicing physicians in a tertiary care hospital (16), likewise, the study on development and validation of a questionnaire assessing Knowledge, Attitude, and Practices about obesity among obese individuals' also utilized a sample size of 100 participants for psychometric evaluation (20). Therefore, the sample of 102 and 210 participants was considered appropriate in this context, while recognizing that more larger samples could further strengthen the stability and generalizability of the model in future studies. Used questionnaire the sample represented a substantial proportion of the available pediatric trauma rehabilitation workforce at the study site. Despite the limited population size, confirmatory factor analysis showed acceptable model fit indices and significant factor loadings across domains, supporting the structural validity of the instrument. For the validation phase, a stratified random sampling approach was used to ensure representation across key professional groups involved in pediatric injury care. Strata were defined by professional cadre, including medical doctors, nurses, and rehabilitation professionals (physiotherapists, occupational therapists, prosthetists, orthotists, optometrists, and speech and language therapists). Within each stratum, participants were randomly selected from staff lists in proportion to the size of each professional group.

### Development and validation process of the study tool

The KAP tool was developed through a structured, multi-phase process (Figure 1). Initial items were generated based on a review of the literature and input from a multidisciplinary expert. The content validity index (CVI) was calculated to assess the content validity of the questionnaire. For each item, the item-level CVI (I-CVI) was calculated as the proportion of experts who rated the item as either 3 or 4, divided by the total number of experts who rated the item (17, 21). Items rated as relevant by at least four of the five experts (I-CVI ≥ 0.80) were retained, whereas items with an I-CVI < 0.80 were reviewed based on the experts' feedback and either revised or removed to improve clarity and relevance and reduce redundancy. A Supplementary Data Sheet presenting each expert's individual item ratings is provided as Supporting Material under the file name “CVI_RATING_EXPERTS.” The revised questionnaire was subsequently pilot tested among 10 healthcare providers to assess clarity, comprehensibility, applicability, feasibility, and administration time. Feedback from the pilot testing led to minor wording revisions and the removal of ambiguous items. The item-level changes and decisions from the pilot testing are presented as the Supplementary Table. Subsequently, the revised tool was field-tested in a separate sample of 210 healthcare providers following the initial field testing of 102 providers for EFA. Construct validity was assessed using factor analysis. Consistent with the approach reported by previous validated KAP survey (22), items with factor loadings below 0.40 were considered to have inadequate representation of the underlying construct and were therefore removed from the instrument (23). This process ensured that only items demonstrating acceptable correlations with their respective domains were retained, thereby strengthening the construct validity of the questionnaire (19). To minimize potential bias, participants involved in the pilot testing phase were excluded from the validation sample. Through this systematic refinement process, a valid and reliable instrument was developed to assess healthcare providers' knowledge, attitudes, and practices regarding pediatric injury rehabilitation.

**Figure 1:**
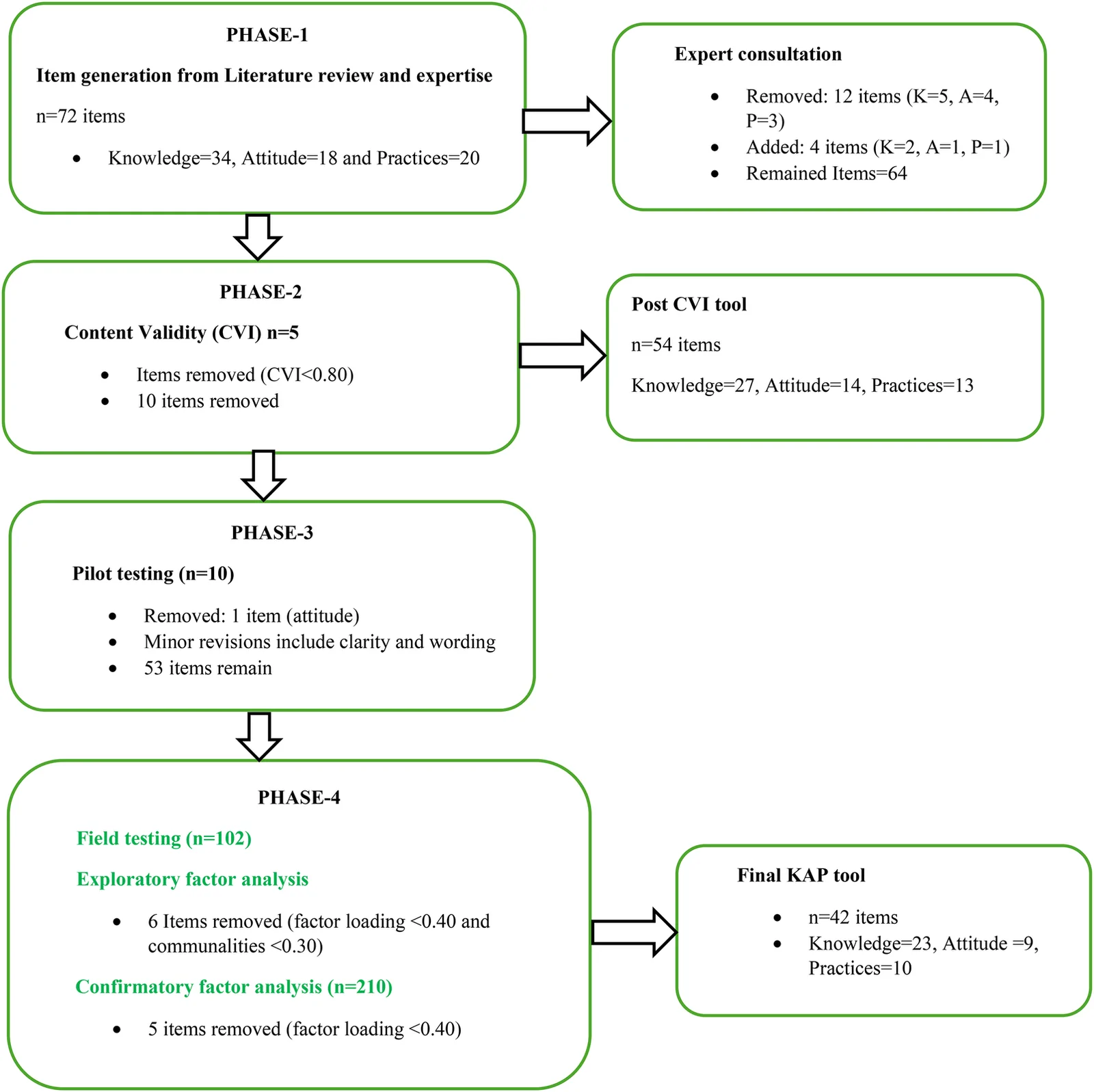
Development and validation process of the KAP tool.

### Item generation and literature review

A structured literature search was conducted to inform the development of questionnaire items by identifying existing KAP studies in healthcare settings. Databases searched included PubMed/MEDLINE, Scopus, and Google Scholar using relevant keywords. Scholar were searched using combinations of the following terms: “knowledge,” “attitude,” “practice,” “rehabilitation,” “pediatric injury,” “pediatric trauma,” “injury in children,” “healthcare providers,” “questionnaire,” “development,” “validation,” “psychometric,” and “healthcare.” The search was limited to English-language articles published between 2015 and 2025. After removing duplicates, 21 articles were screened by title and abstract by two independent reviewers.

Based on predefined inclusion criteria focusing on KAP studies conducted among healthcare providers, 12 full-text articles were assessed. Among these, five studies specifically addressed rehabilitation-related KAP among healthcare providers and were used to inform item generation for the tool. The remaining seven studies, which reported validated KAP instruments had no direct rehabilitation focus, were retained to guide methodological structure and provide a basis for comparison of development and validation approaches. The instrument included objective multiple-choice questions and Likert-scale items to assess healthcare professionals' knowledge, attitudes, and practices related to pediatric injury rehabilitation. The knowledge domain was assessed using objective multiple-choice questions, with one best answer and an “*I don’t know”* option for each item. This approach was used to assess respondents' factual knowledge of key concepts related to pediatric injury rehabilitation rather than their perceived knowledge or confidence. Each correct response was assigned a score of one, while incorrect and “I don’t know” responses were assigned a score of zero. The attitude and practice domains were assessed using Likert-scale items, with response options ranging from strongly disagree to strongly agree. This approach allowed knowledge to be assessed objectively, while attitudes and self-reported practices were measured using standardized response categories that captured participants' perceptions, beliefs, and reported behaviours related to pediatric injury rehabilitation. The full selection process is summarized in Supplementary Table.

### Data analysis

Data analysis was conducted sequentially according to the stages of questionnaire development and validation. All statistical analyses were performed using Stata version 18, while the content validity index (CVI) was calculated using Microsoft Excel spreadsheet.

### Expert content validation

The initially developed questionnaire was evaluated by a panel of five experts. Each expert rated the relevance of individual items using a four-point scale. The item-level content validity index (I-CVI) was calculated as the proportion of experts who rated an item as either 3 or 4. An I-CVI of ≥0.80 was considered acceptable (24). Items with an I-CVI below 0.80 were reviewed based on the experts' comments and were either revised or removed depending on the identified concern and the relevance of the item to the intended construct. The expert ratings and item-level decisions are provided as the Supplementary Material.

### Pilot testing

Following expert content validation and revision of the questionnaire, the revised questionnaire was pilot tested among 10 healthcare providers. The pilot testing assessed clarity, comprehensibility, relevance, applicability, feasibility, and administration time. Feedback from the pilot participants was reviewed to identify unclear or ambiguous items, which were revised or removed before field testing.

### Field testing and descriptive analysis

The revised questionnaire was first field-tested among 102 healthcare providers involved in the rehabilitation management of children following injury, and the data were used for EFA. A separate sample of 210 healthcare providers was subsequently recruited for CFA. Participant characteristics and questionnaire responses were summarized using frequencies and percentages for categorical variables and medians with interquartile ranges (IQR) for continuous variables. Item-level response patterns and inter-item correlations were examined using Pearson or Spearman correlation coefficients, as appropriate. Items considered for removal were evaluated based on their statistical performance, conceptual relevance, and contribution to the overall questionnaire structure.

### Internal consistency

Internal consistency was assessed separately for the knowledge, attitude, and practice domains using Cronbach's alpha, McDonald's omega, and composite reliability. A reliability coefficient of ≥0.70 was considered acceptable (25).

### Exploratory factor analysis

Exploratory factor analysis was conducted using data from 102 healthcare providers to examine the underlying factor structure of the questionnaire. Sampling adequacy was assessed using the Kaiser–Meyer–Olkin (KMO) test, while Bartlett's test of sphericity assessed the suitability of the correlation matrix for factor analysis. Principal axis factoring with oblique rotation was used because the factors were expected to be correlated. The number of factors was determined using eigenvalues >1, scree plot inspection, and parallel analysis (Figures 2, 3 and 4). Factor loadings ≥0.40 and communalities ≥0.30 were considered acceptable. Items with low loadings or communalities were reviewed based on their statistical performance and conceptual relevance before removal. The resulting factor structure was then assessed by CFA using a separate sample of 210 healthcare providers.

**Figure 2:**
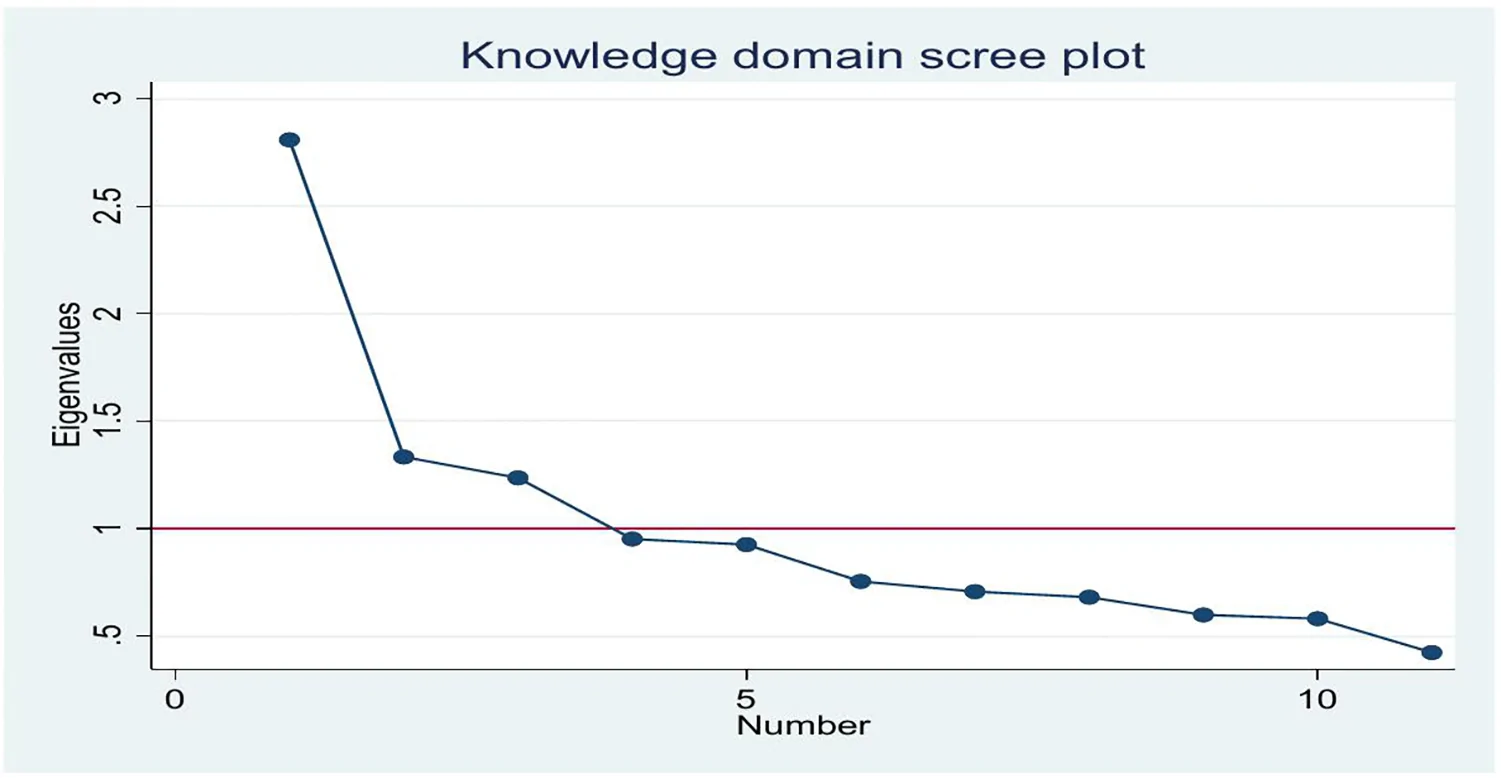
Scree plot showing three factors with eigenvalues greater than 1 in the knowledge domain.

**Figure 3:**
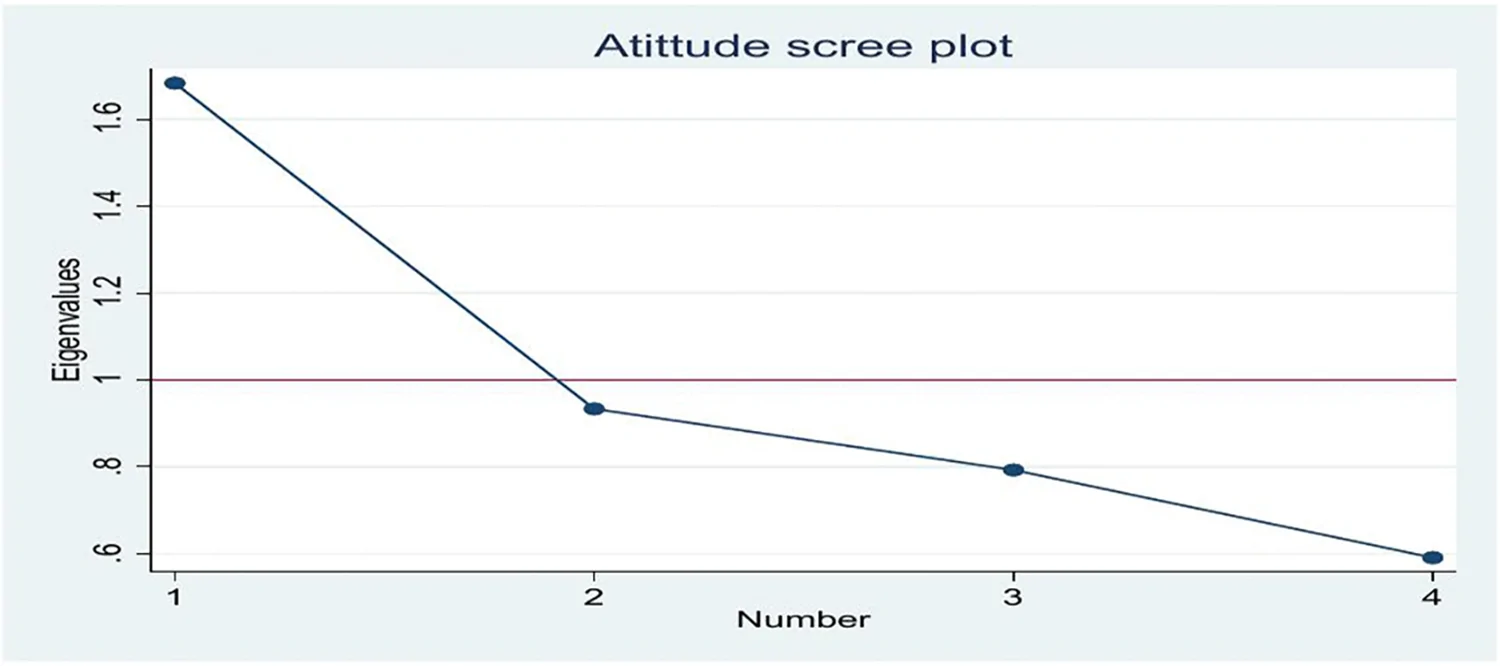
Scree plot showing one factor with an eigenvalue greater than 1 in the attitude domain.

**Figure 4:**
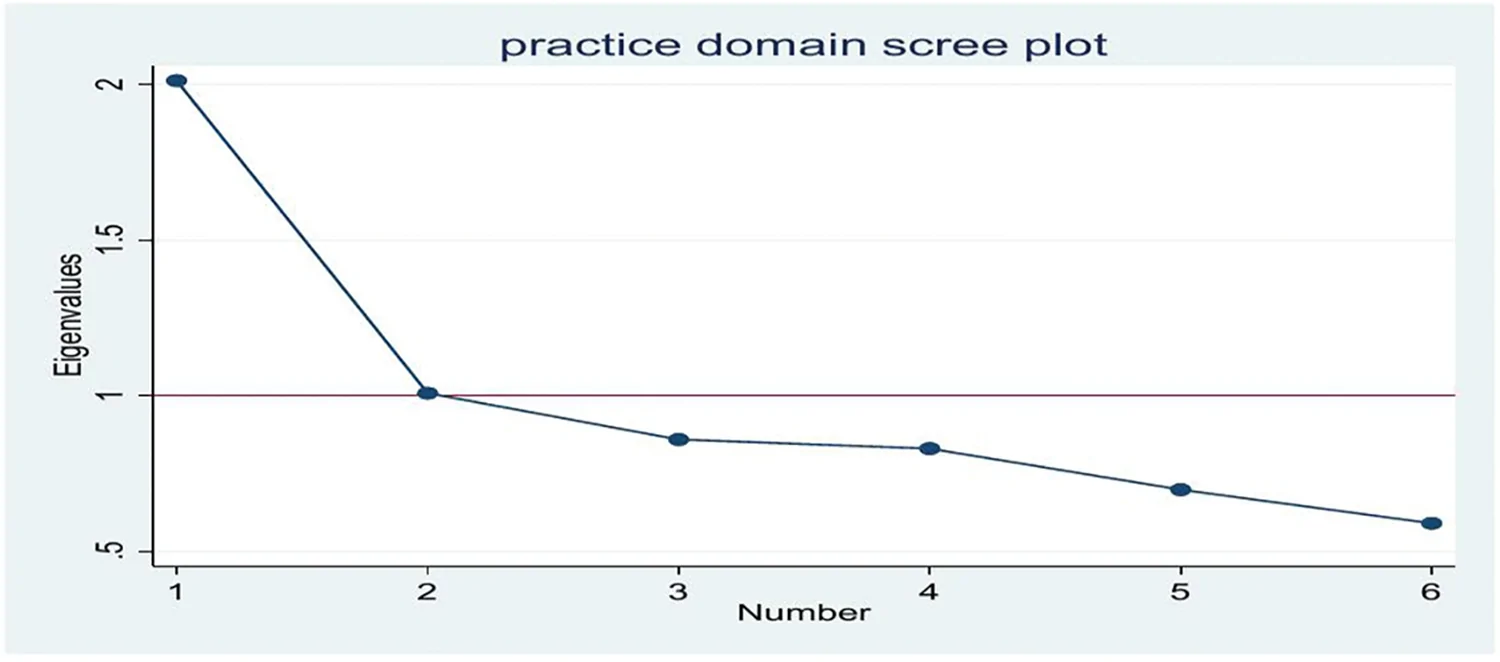
Scree plot showing one factor with an eigenvalue greater than 1 in the practice domain.

### Confirmatory factor analysis and construct validity

CFA was conducted in a separate sample of 210 healthcare providers to evaluate the factor structure identified through EFA. CFA was performed in Stata version 18 using the “***sem*”** command with the weighted least squares mean and variance adjusted (WLSMV) estimator, which is appropriate for ordinal Likert-scale data. Model fit was assessed using the root mean square error of approximation (RMSEA), comparative fit index (CFI), Tucker–Lewis index (TLI), and standardized root mean square residual (SRMR). RMSEA < 0.08, CFI and TLI > 0.90, and SRMR < 0.08 were considered indicative of acceptable model fit (26). Convergent validity was assessed using average variance extracted (AVE), while discriminant validity was assessed using the Fornell–Larcker criterion. Missing data were minimal, and complete-case analysis was therefore used.

### Ethics statement

Ethical approval for this study was obtained from the KCMC University Research Ethics and Review Committee (KUREC) in Moshi, Tanzania (Reference No. KCMC/P.I/Vol.XI/2658). Additional approval was granted by the National Health Research Ethics Committee (NatHREC) of the National Institute for Medical Research (NIMR), Tanzania (Reference No. NIMR/HQ/R.8c/Vol.I/3188). Administrative permission to conduct the study was obtained from the KCMC hospital administration and the heads of the relevant departments. Written informed consent was obtained from all participants before enrolment in the study. Prior to providing consent, participants received detailed information about the study objectives, procedures, potential risks and benefits, confidentiality measures, and their right to withdraw from the study at any time without any consequences. Participants were given adequate time to read the information sheet and ask questions before making a decision regarding participation. Those who voluntarily agreed to participate signed a written consent form, which was documented and securely retained by the research team. A copy of the signed consent form was also provided to each participant for their records. This study involved adult healthcare providers only; therefore, parental or guardian consent was not applicable. Participation was entirely voluntary, and no incentives were provided for participation. To ensure confidentiality, no personal identifiers were collected during data collection. All study data were anonymized and stored in a password-protected database accessible only to authorized members of the research team.

## Results

### Expert feedback and questionnaire refinement

Following the initial development of the questionnaire, the experts provided qualitative feedback on the clarity, wording, organization, and appropriateness of the items during formal content validation. Their feedback informed subsequent refinement of the questionnaire. In the knowledge domain, unclear items were reworded, redundant items were removed, and yes/no response options were changed to Likert scales where appropriate. Some items were revised to better reflect core multidisciplinary pediatric injury rehabilitation concepts. In the attitude domain, unclear wording and inconsistencies were corrected, and some items were reclassified to better reflect their intended meaning. In the practice domain, complex items were simplified, specificity was improved, particularly for referral-related items, and response options were standardized. Overlapping items were removed or merged. Overall, the revisions improved clarity, reduced redundancy, and strengthened the conceptual alignment of items within their intended domains. Detailed expert feedback and the corresponding item-level revisions are provided in Supplementary Table S3.

### Content validity index (CVI) of the study instrument by domain

Content validity was assessed using the item-level Content Validity Index (I-CVI). The knowledge domain (27 items) demonstrated an I-CVI of 0.896, the attitude domain (14 items) an I-CVI of 0.90, and the practice domain (13 items) an I-CVI of 0.815. Overall, the scale-level CVI (S-CVI) for the 54-item instrument was 0.878, indicating strong content validity (Table 1).

**Table 1:** Validity index of the KAP tool by domain (*N* = 5).

Domain	Number of items	Average CVI
Knowledge	27	89.6
Attitude	14	90.0
Practice	13	81.5
Overall Scale	54	87.8

### Pilot testing findings

The pilot study involving 10 healthcare providers assessed the clarity, relevance, and feasibility of the KAP survey. Participants completed the questionnaire within 15–25 min, indicating acceptable feasibility. Minor wording adjustments were made across items in all domains to improve clarity, and instructions were refined for consistency. One attitude item was removed due to consistent misunderstanding. These revisions improved the clarity and usability of the instrument in preparation for field validation (Supplementary Table S4).

### Field testing

#### Socio-demographic characteristics of study participants

EFA sample: A total of 102 healthcare providers were included in the EFA sample. The median age was 27 years, with 57 (55.9%) participants aged 25–34 years. Regarding education, 45 (44.1%) held a bachelor's degree, while nurses comprised the largest professional group, 50 (49.0%). Most participants, 78 (76.5%), had five or fewer years of work experience (Table 2a).

**Table 2a:** Socio-demographic characteristics of healthcare providers included in the EFA sample (*N* = 102).

Variable	Frequency	Percentage
Age in years, *N* (%)		
18–24	27	26.5
25–34	57	55.9
35–44	13	12.7
45–54	5	4.9
Sex		
Male	54	52.9
Female	48	47.1
Residence		
Rural	15	14.7
Urban	65	63.7
Semi-urban	22	21.6
Highest education level		
Certificate	2	1.9
Ordinary diploma	42	41.2
Advance diploma	7	6.9
Bachelor degree	45	44.1
Master	6	5.9
Cadre		
Nurse	50	49.0
Assistant Medical Officer	3	2.9
Medical doctor	21	20.6
Occupational therapist	7	6.9
Prosthetist/Orthotist	5	4.9
Physiotherapist	13	12.8
Optometrist	3	2.9
Years of work experience		
≤5	78	76.5
>5	24	23.5
Marital status		
Single	66	64.7
Monogamous marriage	26	25.5
Cohabiting	3	2.9
Engaged/Dating	7	6.9
Family member with disability		
Yes	42	41.2
No	60	58.8

Age: Median (IQR) 27 (24, 32.5); Years of work experience: Median (IQR) 3 (2,5).

CFA sample: A separate sample of 210 healthcare providers was included in the CFA. The median age was 30 years (IQR: 25–33), with most participants, 125 (59.5%), aged 25–34 years. Slightly more than half were female, 116 (55.2%), and 132 (62.9%) resided in urban areas. Nearly half, 93 (44.3%), held a bachelor's degree or higher. Nurses, 91 (43.3%), and medical doctors, 67 (31.9%), comprised the largest professional groups. Most participants, 151 (71.9%), had five or fewer years of work experience, with a median of 3 years (IQR: 2–6) (Table 2b).

**Table 2b:** Socio-demographic characteristics of healthcare providers included in the CFA sample (*N* = 210).

Characteristic	Frequency	Percentage
Age in years		
18–24	42	20.0
25–34	125	59.5
35–44	30	14.3
45–54	13	6.2
Sex		
Male	94	44.8
Female	116	55.2
Residence		
Rural	33	15.7
Urban	132	62.9
Semi-urban	45	21.4
Highest education level		
Certificate	3	1.4
Ordinary diploma	81	38.6
Advanced diploma	14	6.7
Degree	93	44.3
Master	19	8.0
Cadre		
Nurse	91	43.3
Medical doctor	67	31.9
Occupational therapist	10	4.8
Prosthetist/Orthotist	13	6.2
Physiotherapist	14	6.7
Others	15	7.1
Year experience in working		
≤5	151	71.9
>5	59	28.1
Marital status		
Single	139	66.2
Monogamous marriage	54	25.7
Cohabiting	5	2.4
Engaged/Dating	12	5.7
Family member with disability		
Yes	90	42.6
No	120	57.4

Age in years: Median (IQR); 30 (25–33), Years of experiences: Median (IQR); 3 (2–6).

#### Assessment of suitability for exploratory factor analysis

The suitability of the data for exploratory factor analysis was confirmed through the Kaiser–Meyer–Olkin (KMO) measure and Bartlett's test of sphericity. The KMO values indicated good sampling adequacy across all domains, with values of 0.89 for knowledge, 0.81 for attitude, and 0.78 for practice. Bartlett's test of sphericity was statistically significant for all domains (knowledge: *χ*^2^ = 1,465.5, df = 351, *p* < 0.001; attitude: *χ*^2^ = 239.7, df = 78, *p* < 0.001; practice: *χ*^2^ = 374.0, df = 78, *p* < 0.001). The retained factors explained 79%, 62%, and 53% of the total variance for the knowledge, attitude, and practice domains, respectively, confirming sufficient inter-item correlations and supporting the appropriateness of conducting factor analysis (Table 3).

**Table 3:** Assessment of suitability for exploratory factor analysis (*N* = 102).

Domain	Number of items	KMO	Bartlett *×*^2^	df	Cumulative variance	*p*-value
Knowledge	27	0.89	1,465.5	351	0.79	<0.001
Attitude	13	0.81	239.7	78	0.62	<0.001
Practice	13	0.78	374.9	78	0.53	<0.001

#### Scree plot for KAP domain

The scree plot and eigenvalue criteria supported the retention of three factors for the knowledge domain, with eigenvalues of 2.8, 1.4, and 1.2, respectively. These factors were retained based on eigenvalues greater than one and explained the underlying multidimensional structure of the knowledge domain. In contrast, the attitude and practice domains demonstrated a single-factor structure, with one dominant factor retained in each domain (Figure 2).

#### Exploratory factor analysis and reliability assessment of the KAP instrument

EFA supported the construct validity of the KAP instrument. Items with factor loadings ≥0.40 and communalities ≥0.30 were retained, while poorly performing items were removed. Three factors with eigenvalues greater than one were identified in the knowledge domain, resulting in the retention of 24 items and removal of three items (25, 26, and 27). The retained knowledge items were grouped into three underlying factors, reflecting distinct dimensions of knowledge related to pediatric injury rehabilitation. In the attitude domain, 11 items were retained after excluding items 12 and 13, while 12 practice items were retained following the removal of item 10. The retained factors demonstrated acceptable to good internal consistency, the three factors identified within the knowledge domain showed Cronbach's alpha coefficients of 0.87, 0.79, and 0.77, respectively. The attitude and practice domains demonstrated acceptable internal consistency, with Cronbach's alpha coefficients of 0.78 and 0.76, respectively (Table 4).

**Table 4:** Exploratory factor analysis and reliability assessment of the KAP instrument (*N* = 102).

Domain	Factor	Item	Factor loading	Communalities	Decision	Cronbach *α*
Knowledge	**F1**: Fundamental Principles and Multidisciplinary Rehabilitation Concepts	K1	0.75	0.71	Retained	0.87
		K2	0.77	0.71	Retained	
		K3	0.71	0.64	Retained	
		K4	0.79	0.72	Retained	
		K5	0.76	0.69	Retained	
	**F2**: Physical Rehabilitation, Mobility, and Assistive Technologies	K6	0.72	0.64	Retained	0.79
		K7	0.78	0.71	Retained	
		K8	0.82	0.79	Retained	
		K9	0.77	0.73	Retained	
		K10	0.81	0.78	Retained	
		K11	0.71	0.64	Retained	
		K12	0.79	0.71	Retained	
		K13	0.86	0.82	Retained	
		K14	0.72	0.69	Retained	
		K15	0.75	0.76	Retained	
		K16	0.81	0.79	Retained	
	**F3**: Specialized Rehabilitation Services and Functional Recovery	K17	0.88	0.82	Retained	0.77
		K18	0.79	0.78	Retained	
		K19	0.77	0.71	Retained	
		K20	0.88	0.86	Retained	
		K21	0.76	0.79	Retained	
		K22	0.81	0.78	Retained	
		K23	0.89	0.81	Retained	
		K24	0.73	0.71	Retained	
		K25	0.34^a^	0.26	Removed	
		K26	0.33^a^	0.28	Removed	
		K27	0.29^a^	0.26	Removed	
Attitude		A1	0.69	0.64	Retained	0.78
		A2	0.74	0.69	Retained	
		A3	0.73	0.66	Retained	
		A4	0.70	0.68	Retained	
		A5	0.66	0.63	Retained	
		A6	0.78	0.76	Retained	
		A7	0.86	0.82	Retained	
		A8	0.79	0.78	Retained	
		A9	0.72	0.68	Retained	
		A10	0.57	0.54	Retained	
		A11	0.80	0.79	Retained	
		A12	0.31^a^	0.29	Removed	
		A13	0.32^a^	0.23	Removed	
Practice		P1	0.71	0.67	Retained	0.76
		P2	0.82	0.79	Retained	
		P3	0.64	0.57	Retained	
		P4	0.68	0.63	Retained	
		P5	0.73	0.69	Retained	
		P6	0.72	0.71	Retained	
		P7	0.63	0.59	Retained	
		P8	0.80	0.78	Retained	
		P9	0.78	0.72	Retained	
		P10	0.34^a^	0.28	Removed	
		P11	0.75	0.72	Retained	
		P12	0.74	0.72	Retained	
		P13	0.77	0.74	Retained	

a Removed item; Eigenvalues for knowledge domain: F1 = 2.8, F2 = 1.4, F3 = 1.2; Overall Cronbach *α* = 0.81.

#### Convergent and discriminant validity

The Average Variance Extracted (AVE) values for the constructs were 0.73 for Knowledge, 0.70 for Attitude, and 0.67 for Practice, indicating adequate convergent validity for all constructs. Discriminant validity assessed using the Fornell–Larcker criterion showed that the square roots of AVE were 0.86 for Knowledge, 0.82 for Attitude, and 0.80 for Practice, all of which were greater than the corresponding inter-construct correlations, which ranged from 0.59 to 0.66 (Table 5).

**Table 5:** Convergent and discriminant validity.

Construct	AVE	√AVE	Knowledge	Attitude	Practice
Knowledge	0.73	0.86	0.86	0.61	0.59
Attitude	0.70	0.82	0.61	0.82	0.66
Practice	0.67	0.80	0.59	0.66	0.80

#### Confirmatory factor analysis model fit indices by domain

Confirmatory factor analysis demonstrated an acceptable model fit across all three KAP domains. The knowledge domain, consisting of 23 retained items, showed good model fit with *χ*^2^/df = 1.76, RMSEA = 0.043 (90% CI: 0.014–0.071), CFI = 0.92, TLI = 0.91, and SRMR = 0.077. The attitude domain, comprising 9 retained items, also demonstrated satisfactory model fit with *χ*^2^/df = 1.98, RMSEA = 0.049 (90% CI: 0.017–0.068), CFI = 0.93, TLI = 0.94, and SRMR = 0.079. Similarly, the practice domain with 10 retained items showed acceptable model fit with *χ*^2^/df = 1.64, RMSEA = 0.039 (90% CI: 0.016–0.067), CFI = 0.91, TLI = 0.90, and SRMR = 0.076. Overall, the CFA findings supported the construct validity of the final KAP instrument, demonstrating adequate fit between the hypothesized factor structures and the observed data across the knowledge, attitude, and practice domains (Table 6).

**Table 6:** Confirmatory factor analysis model fit indices by domain (*N* = 210).

Domain	No of items	*X*^2^/df	RMSEA (90% CI)	CFI	TLI	SRMR	CD
Knowledge	23	1.76	0.043 (0.014,0.071)	0.92	0.91	0.077	0.95
Attitude	9	1.98	0.049 (0.017,0.068)	0.93	0.94	0.079	0.83
Practice	10	1.64	0.039 (0.016,0.067)	0.91	0.90	0.076	0.96

RMSEA, root mean square error of approximation; CFI, Comparative Fit Index; TLI, Tucker–Lewis Index; SRMR, standardized root mean square residual; CD, coefficient of determination.

#### Internal consistency reliability of the retained KAP domains

The final instrument demonstrated good internal consistency reliability across all domains. The knowledge domain (23 items) showed good reliability, with a Cronbach's alpha of 0.88 and McDonald's omega of 0.89. The attitude domain (9 items) also demonstrated good internal consistency, with a Cronbach's alpha of 0.81 and McDonald's omega of 0.83. Similarly, the practice domain (10 items) exhibited acceptable reliability, with a Cronbach's alpha of 0.78 and McDonald's omega of 0.79 (Table 7).

**Table 7:** Internal consistency reliability of the retained KAP domains.

Domain	Items	Cronbach *α*	McDonald's *ω*	Composite reliability
Knowledge	23	0.88	0.89	0.91
Attitude	9	0.81	0.83	0.83
Practice	10	0.78	0.79	0.81

Overall: Cronbach *α* = 0.83; McDonald's *ω* = 0.85.

## Discussion

This study developed and evaluated a Knowledge, Attitude, and Practice (KAP) survey tool for assessing healthcare providers involved in the rehabilitation of pediatric injuries in Northern Tanzania. Overall, the final instrument demonstrated satisfactory psychometric properties, providing evidence of content validity, construct validity, and internal consistency for assessing core knowledge, attitudes, and practices relevant to pediatric injury rehabilitation. The instrument demonstrated good internal consistency across all domains following item refinement. Items with low item total correlations, redundancy, or unclear wording were removed, resulting in improved coherence within the respective domains. These findings suggest that clearly defined and domain-specific items enhance reliability, consistent with previous KAP studies (7).

The reliability observed in this study is comparable to that reported in other KAP instruments used in healthcare settings, including Multi-country studies in Sub-Saharan Africa which demonstrated acceptable internal consistency across its domains consistent with prior validation studies in healthcare populations (8). Overall, these findings indicate that the instrument meets recommended psychometric standards for reliability, consistent with prior validation studies in healthcare populations (27).

Content validity results indicated that the items were relevant and appropriately represented the intended constructs. Following expert review, unclear and overlapping items were removed, resulting in CVI values within acceptable ranges across all domains (28). Although slightly lower than values reported in some studies, such as the KAP-Health questionnaire in Brazil (29) and the KAP tool assessing awareness of exercise-based digital games among high school students in Malaysia (22), the findings were within the acceptable range and demonstrate satisfactory content validity. Similar findings have been reported in tools assessing methamphetamine use to adolescents aged 13–17 years residing in high risk substance use zones in central Malaysia (30) and gout patients in Pulau Pinang, Malaysia (31), further supporting the relevance and representativeness of the items included in our tool. These findings are consistent with previous KAP instrument development studies, which have demonstrated that systematic item development and expert evaluation can provide evidence that questionnaire items adequately represent the intended constructs. The alignment with the core domains was established during the initial development of the item pool and further supported through expert evaluation of item relevance, clarity, comprehensiveness, and appropriateness. The application of exploratory factor analysis (EFA) provided important insights into the construct validity and dimensional structure of the instrument. The predefined retention criteria, including factor loadings ≥0.40 and communalities ≥0.30, ensured a rigorous item selection process (32). The KMO values indicated adequate sampling suitability across all domains (0.89 for knowledge, 0.81 for attitude, and 0.78 for practice), with significant Bartlett's tests (*p* < 0.001) confirming the appropriateness of the data for factor analysis. Similar findings were reported in the validation of a KAP instrument assessing occupational accident prevention among rice farmers, where EFA demonstrated acceptable sampling adequacy and supported the underlying factor structure (33). The factor analysis therefore provided evidence supporting the structural validity of the instrument, with the identified factor structures broadly reflecting the conceptual domains of knowledge, attitudes, and practices (34).

The CFA results provided further evidence regarding the fit of the factor structures identified through EFA, demonstrating acceptable model fit across the domains. Similar findings have been reported by Zhang et al. , who observed strong structural validity and reliability for a KAP tool in a healthcare setting (35), supporting the potential utility of our instrument in a comparable healthcare context. These findings are also consistent with other recent studies testing KAP tools in various healthcare areas. For instance, previous research has demonstrated that well-designed KAP instruments can achieve strong CFA fit and high content validity (36).

Similar results have been observed in studies assessing KAP in areas such as pressure injury prevention and other clinical practices, where well-constructed tools demonstrated adequate fit and accurately measured intended constructs (37), Although some studies have reported slightly higher fit indices (38), the results of the present study fall within commonly accepted thresholds, supporting the adequacy of the proposed factor structures in this study sample. Overall, these findings indicate that the instrument provides a valid and reliable measure of healthcare providers' knowledge, attitudes, and practices, consistent with established KAP tools in comparable settings (39).

This study addresses a critical gap by providing a context-specific instrument for assessing core rehabilitation-related knowledge, attitudes, and practices among healthcare providers involved in the rehabilitation management of children following injury in a low-resource setting. The tool provides a standardized measure that can support research, training evaluation, and monitoring of rehabilitation service improvement initiatives. In clinical practice, the instrument can help identify specific gaps in knowledge, attitudes influencing care delivery, and variations in rehabilitation practices. These insights can inform targeted training, support professional development, and strengthen multidisciplinary care to improve the quality of pediatric rehabilitation services. Furthermore, the tool may facilitate assessment of changes following capacity building interventions and contribute to evidence-based planning of rehabilitation services.

## Strengths and limitations

A key strength of this study was the systematic and sequential approach to instrument development and validation, including literature-informed development, expert content evaluation, pilot testing, field testing, EFA, CFA, and assessment of reliability and construct validity. The use of separate samples for EFA and CFA strengthened the evaluation of the factor structure. The inclusion of healthcare providers from multiple professional cadres also supported the relevance of the instrument to multidisciplinary pediatric injury rehabilitation.

The study was conducted at a single referral hospital, which may limit generalizability to other settings and professional groups. Although the expert panel provided complementary methodological, clinical, and rehabilitation perspectives, some rehabilitation disciplines were not represented, which may have limited the breadth of perspectives during content validation. In addition, the instrument assesses core knowledge, attitudes, and practices relevant to rehabilitation management of children following injury rather than profession-specific competencies and should therefore not be interpreted as a comprehensive measure of discipline-specific clinical competence.

Overall, the study provides evidence supporting the content validity, construct validity, and reliability of the instrument. Further validation in larger and more diverse populations and settings is recommended, including assessment of measurement invariance across professional groups and settings.

## Conclusion

The developed KAP instrument demonstrated satisfactory evidence of content validity, construct validity, and internal consistency, with its factor structure evaluated using exploratory and confirmatory factor analyses. The instrument provides a context-specific measure of core knowledge, attitudes, and practices relevant to the rehabilitation management of children following injury among healthcare providers. It addresses an important gap in assessing multidisciplinary pediatric injury rehabilitation in a resource-limited setting and may support the identification of training needs and rehabilitation service improvement.

## Data Availability

The original contributions presented in the study are included in the article/Supplementary Material, further inquiries can be directed to the corresponding author.
