# Supplementary material for: Development and psychometric validation of a tool to assess healthcare providers' knowledge, attitudes, and practices in pediatric injury rehabilitation in Northern Tanzania
Source: Front Rehabil Sci. 2026 Oct 26;7:1960075. doi: 10.3389/fresc.2026.1960075 (PMC13745695; doi:10.3389/fresc.2026.1960075)
Supplement: Supplementary file 1 [file Datasheet1.docx]

**S1 Table: Summary of Selected Studies on KAP Instruments and Validation Approaches**

| **S/N** | **Author (Year)** | **Paper Title** | **Country/Setting** | **Population** | **DOI** |
| --- | --- | --- | --- | --- | --- |
|  |  | **Papers related to KAP in rehabilitation** | | | |
| 1 | Li G et al. (2024) | Knowledge, attitudes, and practices of healthcare professionals toward rehabilitation of peripheral nerve injury | China | Healthcare professionals | doi:10.1038/s41598-024-71232-6 |
| 2 | Cai X et al. (2024) | Knowledge, attitude, and practice of healthcare professionals toward cognitive dysfunction in Parkinson’s disease and cognitive rehabilitation | China | Physicians and nurses | doi:10.1186/s12909-023-04989-5 |
| 3 | Awotidebe TO et al. (2023) | Nigerian physiotherapists’ knowledge, attitude, and practice of digital physical therapy: a cross-sectional study | Nigeria | Physiotherapists | doi:10.1186/s43161-022-00118-3 |
| 4 | Rop S et al. (2022) | Knowledge, attitudes and practice of clinicians managing chronic pain in a tertiary care facility | Kenya | Clinicians | doi:10.4102/sajp.v78i1.1597 |
| 5 | Farpour HR et al. (2021) | Knowledge, Attitude, and Practice of General Practitioners Toward the Rehabilitation Field and Team Experts in Shiraz, Iran | Iran | General practitioners | 10.5812/semj.105821 |
|  |  | **Validation KAP instruments papers** | | | |
| 1 | Rajhathy et al. (2026) | Knowledge, attitudes, and practices (KAP) of nurses towards wound cleansing: design and evaluation of measurement properties of a questionnaire. | Sweeden | Nurses | doi:10.1016/j.jtv.2025.100967 |
| 2 | Zhang et al. (2025) | Development and reliability testing of an orthopaedic nurses' knowledge, attitudes, and practices (KAP) questionnaire on perioperative nutrition management for older adults with hip fractures | China | Orthopaedic nurses | doi:10.1016/j.ijotn.2025.101217 |
| 3 | Taha et al. (2025) | Development and validation of a questionnaire assessing knowledge, attitude, and practice of snake envenomation among health care providers (KAPSE ‐ HCPQ) in sub‐Saharan African countries. | Sub-Saharan Africa | Healthcare providers | doi:10.1111/tmi.14122 |
| 4 | Lister et al. (2025) | Development and validation of a Knowledge, Attitudes and Practices (KAP) questionnaire for healthcare professionals on environmental sustainability in healthcare in Southern Africa | Southern Africa | Healthcare professionals | DOI: 10.12688/f1000research.157487.2 |
| 5 | Dehghani et al. (2023) | Design and psychometric properties of a tool to assess the knowledge, attitude and practice of health care workers for infodemic management (KAPIM-Tool) | Iran | Healthcare workers | doi:10.1186/s12913-023-09822-9 |
| 6 | Andrade et al. (2020) | Designing and Conducting Knowledge, Attitude, and Practice Surveys in Psychiatry: Practical Guidance. | India | Methodological paper (guidance for researchers conducting KAP surveys) | doi:10.1177/0253717620946111 |
| 7 | Mukhopadhyay et al. (2020) | Development and validation of a tool to assess perceptions and practices regarding hypertension and associated comorbidities among primary health care providers of a rural community in India | India | Healthcare providers | doi:10.4103/ijcm.IJCM_490_19 |

**S2 Table: Summary of expert feedback and actions taken by domain**

| **Domain** | **Key Issues Identified** | **Examples of Expert Input** | **Actions Taken** |
| --- | --- | --- | --- |
| **Knowledge** | - Repetition of items - Unclear wording - Inappropriate yes/no format - Limited content coverage | - “Separate combined questions” - “Use Likert scale instead of yes/no” - “Expand to include multidisciplinary roles” - “Clarify pediatric-specific aspects” | - Rephrased unclear items - Removed redundant questions - Converted items to Likert scale - Added items on rehabilitation roles and pediatric needs |
| **Attitude** | - Ambiguity in wording - Mismatch with response options - Domain misclassification | - “Specify what ‘support’ means” - “Some items reflect knowledge, not attitude” - “Use graded Likert scale” | - Reworded ambiguous items - Reclassified miscategorized items - Standardized Likert response format |
| **Practice** | - Lack of clarity and instructions - Complex or double-barreled questions - Incomplete representation of disciplines - Inconsistent response formats | - “Split complex questions” - “Clarify referral practices” - “Include all rehabilitation professionals” - “Use frequency-based responses” | - Simplified and split items - Added clear instructions - Expanded to include multiple disciplines - Revised response options (e.g., frequency scales) |

**S3 Table: Pilot observations summary**

| **Participant** | **Observations / Issues Identified** | **Actions Taken** |
| --- | --- | --- |
| 1 | 3 knowledge items long; 2 attitude items unclear | Shortened knowledge items; rephrased attitude items |
| 2 | 1 attitude item not clear; 3 knowledge items unclear; 2 practice items lengthy | Removed confusing attitude item; shortened knowledge and practice items |
| 3 | 1 attitude item not clear; 2 knowledge items unclear; 3 practice items complex | Rephrased attitude item; simplified knowledge and practice items |
| 4 | 3 knowledge items long; 1 attitude item unclear | Shortened knowledge items; rephrased attitude item |
| 5 | 3 knowledge items unclear; 1 attitude item not understood; 2 practice items poorly structured | Rephrased unclear items; removed problematic attitude item; simplified practice items |
| 6 | Few items required additional explanation | Added clarifying instructions and rephrased items |
| 7 | 4 knowledge items unclear; 2 practice items unclear | Rephrased knowledge items; clarified practice items |
| 8 | Few items unclear; time-consuming | Simplified wording; shortened questions |
| 9 | 3 knowledge items unclear; 2 practice items poorly structured | Rephrased unclear items; simplified practice items |
| 10 | Few items unclear; required additional explanation | Added clarifications and rephrased items |
