# Supplementary material for: Development and psychometric validation of a tool to assess healthcare providers' knowledge, attitudes, and practices in pediatric injury rehabilitation in Northern Tanzania
Source: Front Rehabil Sci. 2026 Oct 26;7:1960075. doi: 10.3389/fresc.2026.1960075 (PMC13745695; doi:10.3389/fresc.2026.1960075)
Supplement: Supplementary file 2 [file Datasheet2.docx]

**KNOWLEDGE ATITUDE AND PRACTICE SURVEY QUESTIONNAIRE FOR HEALTHCARE PROVIDERS ON REHABILITATION OF INJURIES IN PEDIATRIC POPULATION**

**English version**

**SECTION-ONE (Socio-demographic Characteristics)**

**PARTICIPANT ID number:** _____________________

**1. Age:** _______ (Day ___/___Month ___/___ Year____/ ____/ _____/____)

**2. Gender**

1. Male
2. Female

**3. Current marital Status**

1. Single
2. Cohabiting
3. Monogamous Marriage
4. Polygamous marriage
5. Divorced
6. Separated
7. Widowed
8. Engaged/Dating

**4. Place of residence**

1. Rural
2. Semi urban
3. Urban______________________________

**5. Profession/ occupation**

1. Clinical officer
2. Assistant Medical Officer
3. Medical Doctor
4. Physiotherapist
5. Occupational therapist
6. Nurse
7. Prosthetist Orthotist
8. Audiologist, speech pathologist, Speech therapist
9. Optometrist
10. Social worker
11. Clinical psychologist
12. Other (please mention) ______________________________

**6. Highest level of professional education**

1. Certificate
2. Ordinary Diploma
3. Advance diploma
4. Degree
5. Master
6. PhD

**7. Area of specialty**__________________________________________

**8. Please mention the current Department/Unit in which you work (You can tick more than one)**

1. Outpatient department
2. Surgical department (orthopedic surgery, general surgery, neurosurgery)
3. ENT unit
4. Plastic surgery (incl. burn unit)
5. Eye Department
6. Prosthetic/orthotics unit
7. Physiotherapy unit
8. Occupational therapy unit
9. Speech therapy unit
10. Paediatric department
11. Other, (please mention) ____________________________

**9. For how long have you been in practice?** ________________________

**10. How long have you been working in your current position/rank?** __________________________

**11.** Have you ever had a close friend, colleague or member of your family with physical disability?

1. Yes

2. No

**SECTION TWO (Knowledge)**

Rehabilitation professionals are healthcare providers who help individuals recover and improve their ability to function after an injury or illness. Which of the following do you consider as rehabilitation professionals? (Select all that apply)

1.	Physiotherapists

2.	Occupational therapists

3.	Speech and language therapists

4.	Optometrists

5.	Social workers

6.	Psychologists

7.	Prosthetists and orthotists

8.	Chiropractors

9.	Dermatologists

10.Other (Please specify): ______________________

| **S/No.** | **Knowledge question** | **Response options** | **Correct answer** |
| --- | --- | --- | --- |
| **1** | What is the main goal of rehabilitation for children after a major injury? | A. To provide medication only; B. To restore function, independence, and participation in daily activities; C. To replace surgical treatment; D. To keep the child in bed until complete healing; E. I don't know | **B** |
| **2** | When should rehabilitation for a child with a major injury generally begin? | A. Only after complete healing; B. Only after hospital discharge; C. As early as clinically appropriate; D. After one year; E. I don't know | **C** |
| **3** | Which assessment is an important part of rehabilitation management after pediatric injury? | A. History-taking and physical examination; B. Blood test only; C. X-ray only; D. Medication review only; E. I don't know | **A** |
| **4** | What is one important benefit of early rehabilitation following pediatric injury? | A. It prevents all complications; B. It supports functional recovery and may reduce complications associated with inactivity; C. It eliminates the need for medical treatment; D. It guarantees complete recovery; E. I don't know | **B** |
| **5** | Which intervention can help prevent muscle weakness during recovery from injury? | A. Prolonged bed rest; B. Appropriate therapeutic exercise and muscle strengthening; C. Avoiding movement completely; D. Continuous immobilization; E. I don't know | **B** |
| **6** | What is an important purpose of an orthosis in a child recovering from a musculoskeletal injury? | A. To treat infection; B. To provide support, improve alignment, prevent or manage deformity, and support function; C. To replace all rehabilitation exercises; D. To replace surgery in every case; E. I don't know | **B** |
| **7** | How should an orthotic device be selected for a child after injury? | A. The same device should be used for all children; B. According to the child's condition, physical needs, and functional goals; C. According to age alone; D. According to the child's height alone; E. I don't know | **B** |
| **8** | Which rehabilitation device may be prescribed for a child who has lost a limb because of an injury? | A. Orthosis; B. Prosthesis; C. Hearing aid; D. Cervical collar; E. I don't know | **B** |
| **9** | What is an important purpose of an assistive device, such as a walking aid, in pediatric rehabilitation? | A. To support mobility and participation when clinically indicated; B. To replace all physical activity; C. To be used only by children with permanent disabilities; D. To prevent children from walking independently; E. I don't know | **A** |
| **10** | Which approach can be used as part of rehabilitation to help manage pain following some pediatric musculoskeletal injuries? | A. Appropriate therapeutic exercise and physical modalities; B. Complete bed rest for all children; C. Avoiding all movement; D. No treatment until the pain disappears; E. I don't know | **A** |
| **11** | What is a common rehabilitation use of transcutaneous electrical nerve stimulation (TENS)? | A. Management of some types of pain; B. Healing all bone fractures; C. Correcting limb deformities; D. Treating bacterial infections; E. I don't know | **A** |
| **12** | Which physical modality may be used during the early phase of some acute musculoskeletal injuries to help reduce pain and swelling? | A. Cold therapy; B. Heat therapy; C. Infrared therapy only; D. Continuous ultrasound; E. I don't know | **A** |
| **13** | Which statement best describes the use of heat therapy in rehabilitation? | A. It is appropriate for every acute injury; B. It may be used in selected conditions to help reduce pain and muscle stiffness; C. It is used to treat infections; D. It should replace exercise therapy; E. I don't know | **B** |
| **14** | What is one potential use of hydrotherapy in pediatric rehabilitation? | A. Supporting movement and exercise in water for selected children; B. Treating all types of fractures; C. Replacing all other rehabilitation interventions; D. Treating infections; E. I don't know | **A** |
| **15** | Which rehabilitation professional is primarily trained to assess and manage speech, language, and communication difficulties? | A. Speech and language therapist; B. Orthotist; C. Physiotherapist; D. Prosthetist; E. I don't know | **A** |
| **16** | Following a pediatric head injury, which problem may require assessment by an appropriate eye-care or vision rehabilitation professional? | A. Visual impairment or visual-function problems; B. Muscle weakness only; C. Bone fracture only; D. Speech impairment only; E. I don't know | **A** |
| **17** | Which rehabilitation professional commonly helps children regain skills needed for self-care, play, school activities, and other daily activities after injury? | A. Occupational therapist; B. Radiographer; C. Pharmacist; D. Laboratory scientist; E. I don't know | **A** |
| **18** | Which area may be addressed by occupational therapy in a child recovering from a brain injury? | A. Cognitive and daily living skills; B. Surgical fixation of fractures; C. Treatment of infection with antibiotics; D. Blood transfusion; E. I don't know | **A** |
| **19** | Which rehabilitation professional is commonly involved in improving movement, strength, balance, and mobility after pediatric injury? | A. Physiotherapist; B. Pharmacist; C. Radiographer; D. Laboratory scientist; E. I don't know | **A** |
| **20** | Which intervention may help improve mobility and physical function in a child recovering from injury? | A. Individualized therapeutic exercise and mobility training; B. Prolonged bed rest for every child; C. Avoidance of physical activity; D. Continuous immobilization; E. I don't know | **A** |
| **21** | Which child is most likely to require rehabilitation focused on cognitive and functional recovery? | A. A child with a significant traumatic brain injury; B. A child with a minor superficial skin injury only; C. A child with an uncomplicated minor nail injury; D. A child with no functional limitation; E. I don't know | **A** |
| **22** | Which statement best describes rehabilitation after pediatric major injury? | A. The same rehabilitation programme should be used for every child; B. Rehabilitation should be individualized according to the child's injury, needs, abilities, and functional goals; C. Rehabilitation is only necessary after permanent disability develops; D. Rehabilitation should begin only after hospital discharge; E. I don't know | **B** |
| **23** | What is an important role of physiotherapy in children recovering from serious injury? | A. Improving physical function, movement, mobility, and, when indicated, respiratory function; B. Prescribing all medications; C. Performing surgical procedures; D. Treating infections with antibiotics; E. I don't know | **A** |

**SECTION THREE (Attitude)** **(Tick a choice based on your preference)**

| ***SNO*** |  | ***Strongly Disagree*** | ***Disagree*** | ***Unsure*** | ***Agree*** | ***Strongly Agree*** |
| --- | --- | --- | --- | --- | --- | --- |
| 1. | Do you believe that rehabilitation plays a vital role in the overall recovery and improve quality of life for pediatric injured patients? |  |  |  |  |  |
| 2. | Do you feel confident in working with a multidisciplinary rehabilitation team for the care of pediatric injured patients? |  |  |  |  |  |
| 3. | Are you willing to actively participate in the rehabilitation process to paediatric injured patients with injury to help improve the outcome? |  |  |  |  |  |
| 4. | Do you believe that early initiation of rehabilitation can improve functional outcomes for paediatric injured patients? |  |  |  |  |  |
| 5. | Do you believe rehabilitation is important in the overall care of pediatric injured patients? |  |  |  |  |  |
| 6. | Do you feel confident in your ability to assist and support pediatric injury patients throughout their rehabilitation process? |  |  |  |  |  |
| 7. | Do you think that working together across various healthcare disciplines enhances the effectiveness of rehabilitation services for pediatric injured patients? |  |  |  |  |  |
| 8. | Do you believe that rehabilitation programs can improve the quality of life for pediatric patients with disabilities due to injuries? |  |  |  |  |  |
| 9. | How do you view the collaboration between different healthcare disciplines in providing rehabilitation services for pediatric trauma patients? |  |  |  |  |  |

| **S/NO** | **SECTION FOUR (PRACTICE)** **(Tick a choice based on your preference)** | ***Always*** | ***Often*** | ***Sometimes*** | ***Rarely*** | ***Never*** |
| --- | --- | --- | --- | --- | --- | --- |
| 1. | Have you ever taken personal initiative to refer a pediatric injury patient to any of the rehabilitation department? |  |  |  |  |  |
| 2. | Do you frequently refer pediatric injury patient to rehabilitation services when they are in hospital-based care? |  |  |  |  |  |
| 3. | Do you involve rehabilitation specialists in the treatment planning and care of pediatric injury patients? |  |  |  |  |  |
| 4. | Have you ever received any training or education on rehabilitation services for pediatric injury patients? |  |  |  |  |  |
| 5. | Do you follow-up injury pediatric patients after discharge? |  |  |  |  |  |
| 6.  6a.  6b. | Have you or anyone in your family ever received rehabilitation services?   1. Yes 2. No   If yes, please specify the type of rehabilitation services received (e.g., physical therapy, occupational therapy, speech therapy, prosthetics/orthotics). You can mention more than one if so. _____________________________________  How satisfied were you or your family member with the rehabilitation services received? |  |  |  |  |  |
|  |  | ***Very unsatisfied*** | ***Unsatisfied*** | ***Neither satisfied nor unsatisfied*** | ***Satisfied*** | ***Very satisfied*** |
|  |  |  |  |  |  |  |
| 7. | What would be your goal to refer pediatric injury patients for rehabilitation management? (Rank top five goals when referring patients for rehabilitation).   a) Preventing or treating deformities b) Increasing limb function c) Reducing/preventing/hindering limb weight-bearing d) Pain reduction e) Controlling involuntary movements f) Increasing level of function and activity for a child a whole g) Stimulate cognitive functions h) Reduce spasticity and promote relaxation i) Posture correction and readjustment j) I do not know |  |  |  |  |  |
|  |  | ***I don’t know*** | ***Not important*** | ***Neutral*** | ***Important*** | ***Very important*** |
| 8. | In your opinion how important do you think rehabilitation is for children with disabilities or injuries due to trauma? |  |  |  |  |  |
| 9 | Do you believe that rehabilitation programs can improve the quality of life for pediatric with disabilities or injuries due to trauma? |  |  |  |  |  |
|  |  | ***I don’t know*** | ***Not supportive*** | ***Neutral*** | ***Somewhat supportive*** | ***Very supportive*** |
| 10. | How supportive have you been/ will you being in including pediatric rehabilitation services as part of healthcare systems? |  |  |  |  |  |

After reading and filling out the questionnaire, would you refer pediatric patients to rehabilitation experts?

a) Strongly No
b) No
c) Unsure
d) Yes
e) Strongly Yes
